# Nitrate sources and mixing in the Danube watershed: implications for transboundary river basin monitoring and management

**DOI:** 10.1038/s41598-022-06224-5

**Published:** 2022-02-09

**Authors:** J. Halder, Y. Vystavna, L. I. Wassenaar

**Affiliations:** Department of Nuclear Sciences and Applications, Vienna International Centre, International Atomic Energy Agency, 1400 Vienna, Austria

**Keywords:** Biogeochemistry, Hydrology

## Abstract

Dispersed and unknown pollution sources complicate water management in large transboundary watersheds. We applied stable isotopes of water and nitrate together with contaminants of emerging concern (CECs: carbamazepine, caffeine, sulfamethoxazole, perfluorooctanoic acid and 2,4-dinitrophenol) to evaluate mixing and inputs of water and contaminants from tributaries into the mainstem of the transboundary Danube River. Stable isotope (*δ*^18^O, *δ*^2^H) variations from low values (− 13.3 ‰, − 95.1 ‰) in the Upper Danube after the Inn River confluence to high values (− 9.9 ‰, − 69.7 ‰) at the Danube River mouth revealed snowmelt dominated tributary mixing (~ 70%) in the mainstem. Stable isotopes of nitrate (*δ*^15^N-NO_3_) in the Danube River varied from lower values (+ 6.7 ‰) in the Upper Danube to higher values after the mixing with Morava River (+ 10.5 ‰) and showed that cold snowmelt can reduce biological activity and controls nitrate biotransformation processes in the mainstem up to 1000 km downstream. Data on emerging contaminants affirmed the low biodegradation potential of organic compounds transferred into the mainstem by tributaries. We found pollutant source tracing in large rivers is complicated by mixing of multiple sources with overlapping isotopic signatures, but additional tracers such as CECs improve the interpretation of hydrological processes (e.g., water transit time) and support tracing of nitrate pollution sources, and biogeochemical processes. Our approach can be applied to other watersheds to improve the understanding of dilution and mixing processes. Moreover, it provides directions for improving national and transboundary water quality monitoring networks.

## Introduction

Whereas knowledge about sources of water-borne contaminants, dilution, and mixing processes in rivers is crucial for informing sustainable water management, it remains challenging to obtain reliable information in large transboundary rivers and watersheds due to the large size, dispersed or unknown sources of pollution, and different adaptation measures implemented by various stakeholders in the riparian countries^1^. The Danube River Basin (DRB) (801,463 km^2^ of watershed area) is shared by 19 countries with a population of 81 M and it is the world’s most international watershed and second longest European river (2857 km)^2,3^. The DRB stretches from the Black Forest in Southern Germany to discharge in the Black Sea in Romania and Ukraine, contributing about 40% of the freshwater input into the Black Sea. The flow and hydrochemistry of the Danube River (and most of its tributaries) are significantly impacted by human and land use activities^4,5^. Over the past decade phosphorus loadings significantly decreased via improved wastewater management in the DRB, but nitrate (NO_3_^−^) loadings have persisted despite a decrease in nitrogen (N) surpluses in agriculture practices across the watershed^3,6,7^. The DRB management is coordinated by the *International Commission for the Protection of the Danube River* (ICPDR) which recommended a reduction in N pollution of ground and surface waters by implementation of the European Union (EU) Nitrates Directive (1991)^8^. In addition, EU Member States (MS) must reduce their diffuse N pollution sources by implementation of supplementary agri-environmental measures linked to the EU Common Agricultural Policy and by application of cost-effective beneficial management practices for Non-EU MS. Although wastewater management has improved, sub-standard treatment of urban wastewaters in Danube tributaries remains a major water quality issue in the DRB^9–12^.

To better understand the contribution (mixing and dilution) of water and contaminants from tributaries into the Danube mainstem, we used environmental isotopes along with selected compounds of emerging concern (CECs). Stable hydrogen (*δ*^2^H) and oxygen isotope (*δ*^18^O) ratios in precipitation and runoff are well-established conservative tracers of water origin, mixing, and dilution patterns in global rivers^13^. Nitrogen and oxygen isotope ratios of nitrate (*δ*^18^O-NO_3_, *δ*^15^N-NO_3_) help to delineate NO_3_^−^ sources and biogeochemical cycling processes^14–21^, but challenges remain when NO_3_^−^ sources have overlapping ^15^N isotopic compositions (e.g., sewage and manure)^21^. Additional tracers, such as CECs (pharmaceuticals, detergents, artificial sweeteners, industrial compounds, etc.) are widely detectable in water but are not regulated and may support isotopic data^22,23^. Having direct relationships to sources and known physicochemical properties, CECs are seen as ideal tracers of various anthropogenic activities (industrial wastewater, urban sewage, and agricultural waste)^23–25^. Detailed water quality surveys on large transboundary river basins are rare, and this multi-disciplinary study provides important insights into the transformation of the isotopic and chemical signals that derived from main tributaries to the Danube mainstem. The aim of the study was to evaluate mixing and inputs of water and nitrate from tributaries to the mainstem of the transboundary Danube River and thereby to highlight directions for the improvement of national and transboundary water quality networks and monitoring.

## Results

### Stable isotopes of water in precipitation, Danube mainstem and tributaries

**Figure 1:**
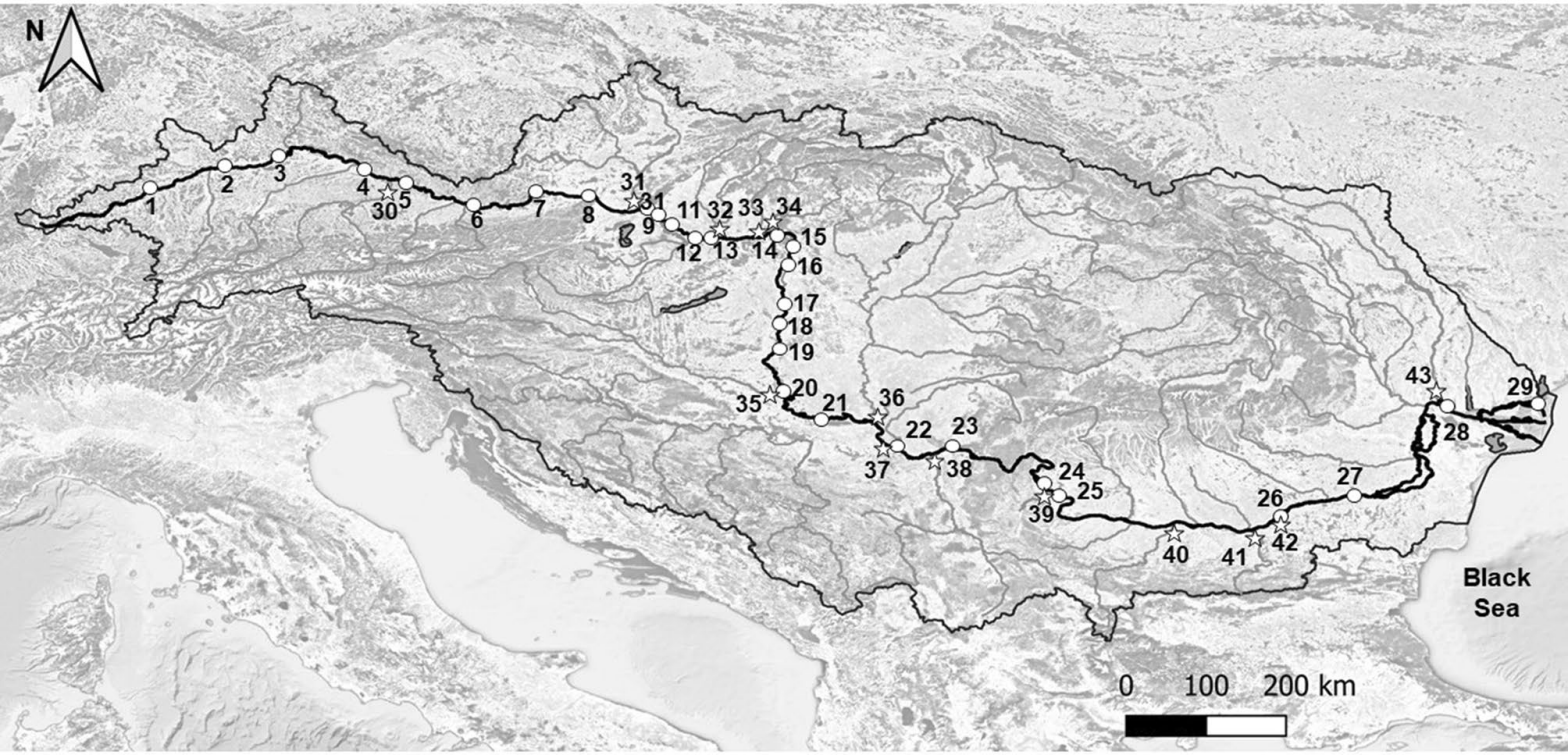
Sampling sites (2019) on the Danube River (1–29) and its tributaries (Inn-30, Morava-31, Vah-32, Hron-33, Ipel-34, Drava-35, Tisza-36, Sava-37, Velika-Morava-38, Timok-39, Iskar-40, Jantra-41, Russenski-Lom-42, Prut-43). Georeferenced data were taken from DanubeGIS (https://www.danubegis.org/) and topographic background from OpenTopoMap.

In total, 43 sites were sampled in the DRB in 2019, including 29 sites along the Danube mainstem and 14 sites at the mouths of main tributaries (Fig. 1).

**Figure 2:**
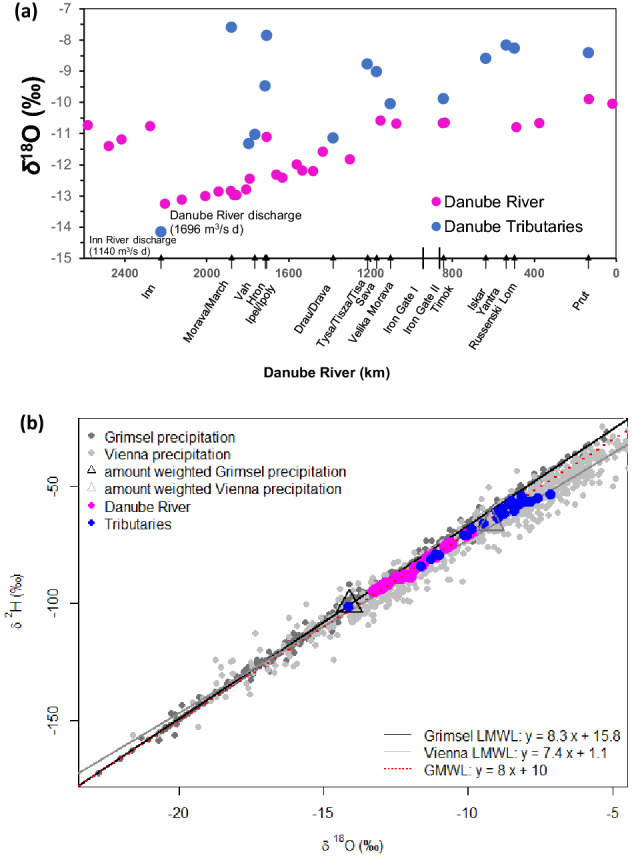
(**a**) Longitudinal isotope values (*δ*^18^O) for the Danube mainstem and tributaries (Fig. 1) and (**b**) *δ*^18^O and *δ*^2^H plot showing isotopic compositions of the Danube mainstem and tributaries in comparison to precipitation inputs exhibited by the Local Meteoric Water Lines (LMWL) for Grimsel and Vienna.

Over the sampling period, average discharge of the Danube River was 1696 m^3^/s (Achleiten-Danube station). Average *δ*^18^O and *δ*^2^H values along the Danube River transect were − 11.7 ‰ and − 83.9 ‰. Minimum *δ*^18^O and *δ*^2^H values (− 13.3 ‰ and − 95.1 ‰) occurred following the confluence of the large alpine Inn River, whereas maximum values (− 9.9 ‰ and − 69.7 ‰) were observed close to the Danube River mouth (Fig. 2a). Isotope mass balance calculations showed that the water fraction of the Inn River after the confluence with the Danube River was about 74% (Table SI-1). This was confirmed by a binary isotope plot (Fig. 2b) where the isotopic composition of the Danube River was close to the amount weighted value of the high elevation (Grimsel) precipitation station (Fig. 2b).

The Danube tributaries had *δ*^18^O and *δ*^2^H variations ranging from − 14.2 and − 101.8 ‰ (Inn River) to − 7.6 ‰ and − 55.2 ‰ (Morava River), respectively, with relatively more positive *δ*^18^O values compared to the Danube mainstem (Figs. 2a, b and SI-1). Water temperatures in the Upper Danube were lower and had a high *d*-excess, indicative for lower evaporative conditions compared to the Middle and Low Danube (Fig. SI-2). The low snowmelt isotopic signal remained constant until the confluence with the Ipel River in the Middle Danube (Fig. 2a). The water isotopic compositions in the Danube River changed towards more positive isotope values following progressive longitudinal mixing with lowland tributaries, reaching the highest values after the confluences of the ^18^O enriched Tisza and Sava River until the Danube River mouth at the Black Sea (Fig. 2a).

### Stable isotopes of nitrate, ions, CECs in Danube mainstem and tributaries

**Figure 3:**
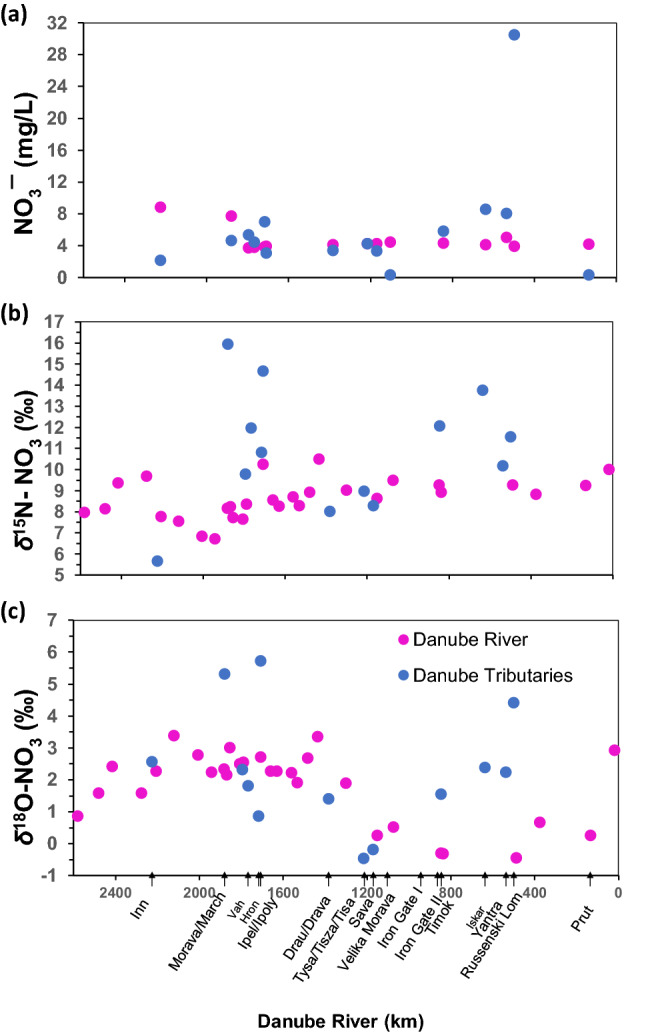
(**a**) NO_3_^−^ (mg/L) concentration, (**b**) *δ*^15^N-NO_3_ and (**c**) *δ*^18^O-NO_3_ values in Danube mainstem and tributaries (x-axis in **c** corresponds to x-axis in **a** and **b**).

The NO_3_^−^ concentrations of the Danube River remained relatively constant after the confluence with the Inn River, however tributaries had highly variable concentrations (Fig. 3a). Like for the stable water isotopes, a significant decrease in the *δ*^15^N-NO_3_ was observed after the confluence of the Inn River (+ 6.7 ‰) until mixing with the Morava River (+ 10.5 ‰) since both tributaries contributed NO_3_^−^ with highly distinctive *δ*^15^N-NO_3_ compositions (Fig. 3b). Thereafter the *δ*^15^N-NO_3_ value remained relatively constant over the last 1300 km downstream (Fig. 3b).

**Figure 4:**
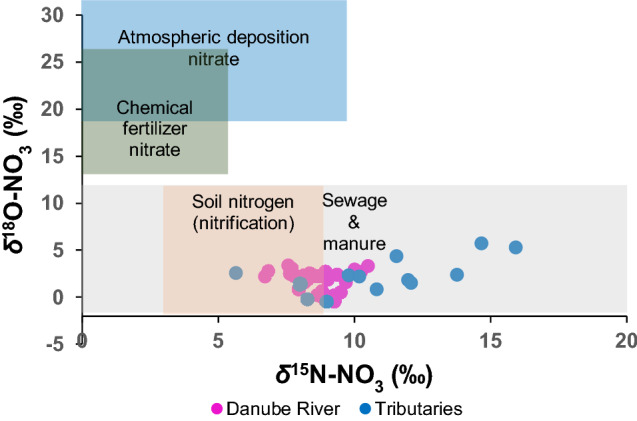
The *δ*^15^N-NO_3_ vs *δ*^18^O-NO_3_ framed within potential nitrate sources according to Matiatos et al.^21^.

The *δ*^18^O-NO_3_ values were higher in the Upper Danube (+ 3.4 ‰), but lower after the confluence with the Tisza and Sava Rivers (− 0.5 ‰) and then remained relatively constant for ca. 400 km despite several tributaries adding NO_3_^−^ with higher *δ*^18^O values along this transect (Fig. 3c). The lower *δ*^15^N-NO_3_ values in the Danube River were in a range similar to natural soil N origins, but the relatively higher *δ*^15^N-NO_3_ values in the tributaries plotted within the typical N isotopic compositions of sewage and animal manure (Fig. 4).

**Figure 5:**
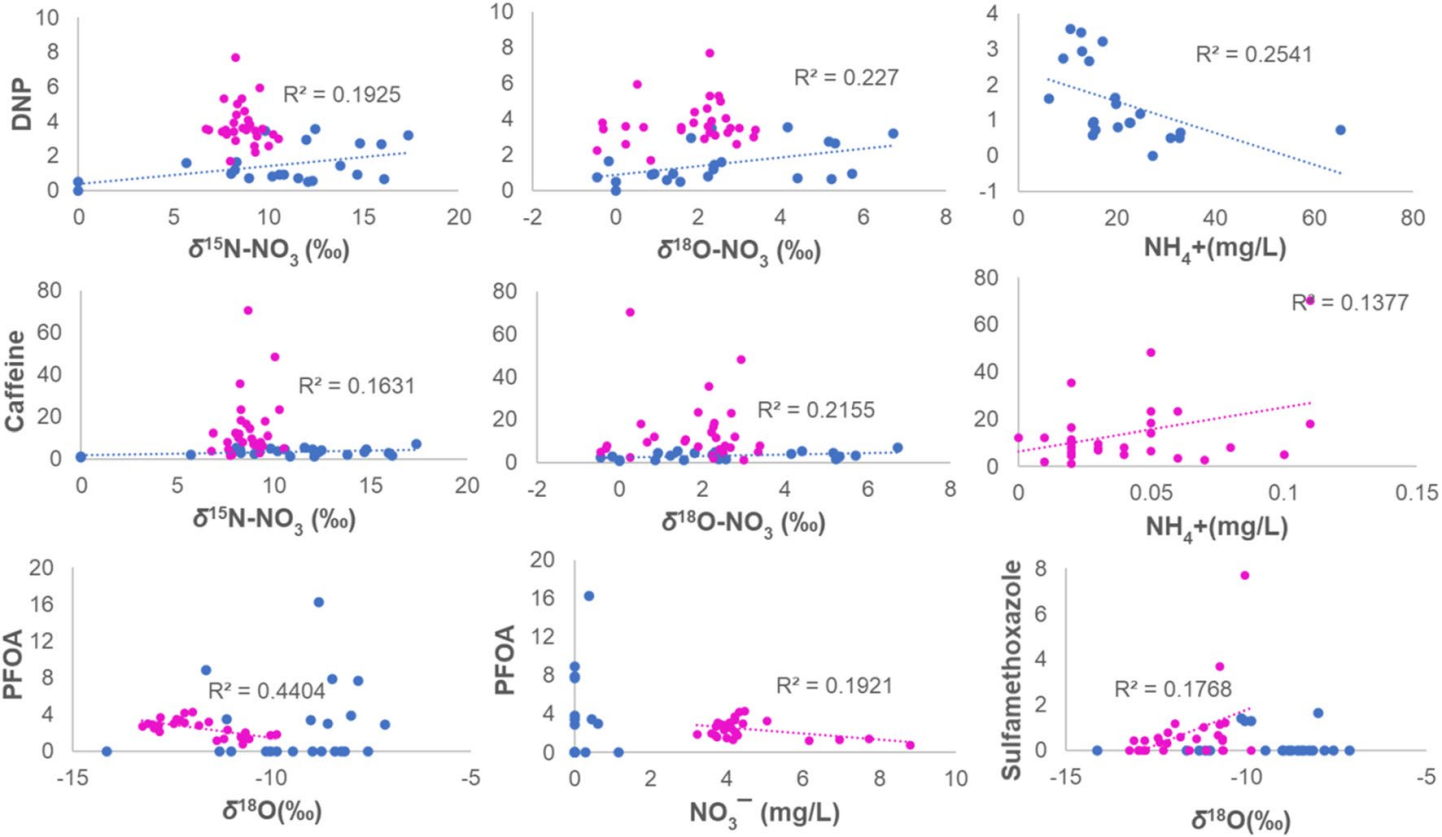
Linear regression plots between CEC, stable isotopes, nitrate, and ammonium in the Danube mainstem (magenta dots) and tributaries (blue dots). Significant (*p* < 0.05) trends are indicated with a coefficient of determination (R^2^).

In the Danube mainstem, the CEC concentrations varied from 1.3 to 70.4 (median $\tilde{x}$ is 9.7) ng/L for caffeine, from 2 to 101 ($\tilde{x}$ = 9.1) ng/L for carbamazepine, from 0 to 7.7 ($\tilde{x}$ = 0.4) ng/L for sulfamethoxazole, from 0.8 to 4.3 ($\tilde{x}$ = 2.5) ng/L for PFOA and from 1.7 to 7.7 ($\tilde{x}$ = 3.5) ng/L for DNP (Tables SI-2 and SI-3). In the tributaries, CECs concentration varied from 1.2 to 28 ($\tilde{x}$ = 10.6) ng/L for caffeine, from 1 to 34 ($\tilde{x}$ = 10.1) ng/L for carbamazepine, from 0 to 8.7 ($\tilde{x}$ = 0.4) ng/L for sulfamethoxazole, from 0 to 3.5 ($\tilde{x}$ = 0.9) ng/L for PFOA and from 1 to 7.2 ($\tilde{x}$ = 3.2) ng/L for DNP (Table SI-3). In the mainstem, *δ*^15^N of NO_3_ had a negative correlation with *δ*^18^O of NO_3_ and a positive correlation with water *δ*^18^O, NH_4_^+^, SO_4_^2−^ (Figs. 5 and SI-3). Nitrate had a positive correlation with sulfamethoxazole and ammonium had a positive correlation with caffeine. PFOA and DNP represented as a separate correlation group (Fig. SI-3). PFOA had a negative correlation with water *δ*^18^O and nitrate (Fig. 5). Sulfamethoxazole had a positive correlation with water *δ*^18^O (Fig. 5).

In the tributaries, *δ*^15^N-NO_3_ and *δ*^18^O-NO_3_ had a positive correlation and plotted with Cl^−^ and CECs in one group (Fig. SI-3). DNP had a negative correlation with ammonium (Fig. 5). Caffeine had a positive correlation with stable isotopes of nitrate (Fig. 5). Ion concentrations were higher in the Danube tributaries than in the mainstem (Fig. SI-4). The higher ammonium concentrations were in the Lower Danube after the Sava River confluence (Fig. SI-5).

## Discussion

Our results show that the origin and mixtures of Danube River water with tributaries have significant local variability, particularly between the Upper and Lower Danube in agreement with earlier studies^26–30^. Tributary contributions to the dilution and mixing within the Danube mainstem have a strong impact on water chemistry, and particularly, on nitrate. Due to the large geographical size of the DRB, multiple NO_3_^−^ sources and modifications of the nitrate isotopic signal before entering the mainstem, it was not possible to clearly identify and quantify specific NO_3_^−^ sources. Based on the *δ*^15^N-NO_3_ values, nitrate is likely exported or discharged into the Danube River from soils with an additional admixture of wastewater or manure from tributaries (Fig. 4). The occurrence and significant correlation of nitrate and its isotopes with CECs suggest that wastewater inputs derived via tributaries is an important admixture source in the Danube River (Table SI-3, Fig. SI-3) confirming earlier studies in the DRB^9,10^ and in other basins worldwide^21,31–33^. The positive correlation of nitrate isotopes with caffeine and DNP showed these CECs are relevant chemical tracers of the sewage inputs (Fig. 5). However, the negative correlation between DNP and ammonium, and positive correlation between caffeine and ammonium (Fig. 5) indicate that DNP is a viable tracer of treated sewage, but caffeine is a tracer of raw sewage inputs into the Danube River.

The contribution of mineralized fertilizers or atmospheric deposition was not evident based on the range of *δ*^18^O-NO_3_ and *δ*^15^N-NO_3_ values in the Danube River. However, previous studies on the DRB^34,35^ indicated that nitrate input by agriculture is substantially reduced by crop uptake, soil denitrification and by riparian filter strips. In other longitudinal river studies^19,20,36^, *δ*^15^N-NO_3_ values in rivers often increase by stream order because headwaters often drain predominantly pristine forested areas, where low nitrate concentrations are derived from natural N_2_-fixation followed by remineralization and nitrification with a *δ*^15^N-NO_3_ < 7 ‰. In the Danube River, such low “natural” *δ*^15^N-NO_3_ values were found in the predominantly forested watersheds of the alpine Inn River (Fig. 3b), and this contribution of soil nitrate was particularly important during spring snowmelt when leaching of soil water from slopes and banks was higher^37^. Since nitrate is produced via nitrification in the soil, the corresponding *δ*^18^O-NO_3_ reflected ambient river water *δ*^18^O and air *δ*^18^O-O_2_ at the time of nitrification. When urban land use increases along the river course, point sources of domestic and industrial wastewaters cumulatively contribute new NO_3_^−^ from municipal sewage, and hence the *δ*^18^O-NO_3_ values generally increase (*δ*^18^O-NO_3_ > 7 ‰). This pattern was clearly seen in the Sava River and it could be assumed that nitrate in the Lower Danube River thereby originates from a combination of soil nitrification and incoming admixtures of NO_3_^−^ from wastewater and manure^37,38^.

There was no evidence for in-situ denitrification since the Danube river was well-oxygenated and thus likely to preserve nitrate (dissolved oxygen > 2 mg/L) (Fig. SI-6). Moreover, *δ*^18^O and *δ*^15^N values of nitrate would both simultaneously increase if there was denitrification, which was not evident in the data analysis (Fig. SI-3). There was no decreasing profile of NO_3_^−^ concentrations in the Danube mainstem. Denitrification within the Danube River sediments or in riparian zones cannot be excluded, but the isotopic signal linked to this process could not detected based on synoptic data. It is also likely that assimilation and nitrification occur in the water column of the Danube River, however no parallel enrichment in *δ*^15^N-NO_3_ and *δ*^18^O-NO_3_ was observed. This is expected to be a seasonal feature and would require higher time–frequency sampling at key points along the Danube River, and ideally covering the winter period with low biological activities^21^. Another possible N removal process is assimilation or uptake of inorganic nitrogen compounds into living organisms via biosynthesis, which has also been observed as a main removal process of nitrate in Central European rivers during summer months^15^.

**Figure 6:**
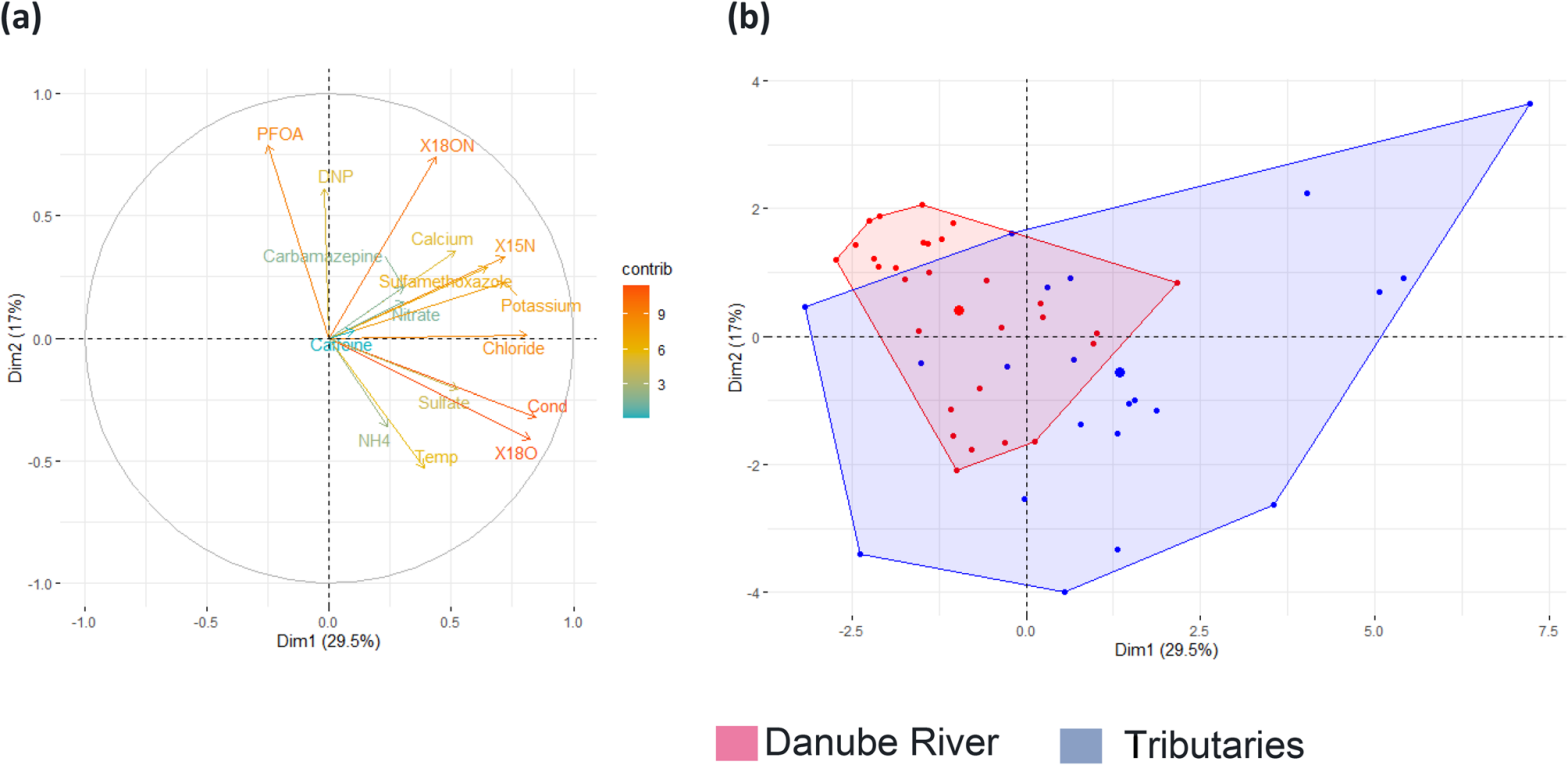
PCA plot depicts the relation between (**a**) water temperature (Temp), electrical conductivity (Cond), ammonium (NH_4_), *δ*^18^O in water (X18O), *δ*^18^O in nitrate (X18ON) and *δ*^15^N in nitrate (X15N), compounds of emerging concern (Caffeine, Carbamazepine, Sulfamethoxazole, PFOA and DNP) and major ions (Potassium, Sulfate, Calcium, Nitrate, Chloride) and (**b**) categories (Danube River and tributaries).

The similar nitrate and isotopic compositions to previous surveys^35,39^ indicated that nitrate sources, processes, and mixing patterns have not changed significantly over this decadal timeframe and leads to the conclusion that nitrate cumulatively originates from diffuse sources like soil nitrate, agriculture or septic tanks and is transported to rivers and tributaries via baseflow/groundwater inputs. Tributaries are clearly transporting wastewater derived CECs and nitrate to the Danube mainstem, but in contrast to ions and stable water isotopes, no peaks in NO_3_^−^ concentrations were observed in the Danube River after the tributary confluences. However, as seen from the shift in the *δ*^18^O-NO_3_, the mixing with the alpine Inn River could reduce the impact of biological processes owing to the colder-water temperatures in the Upper Danube in comparison to downstream (Fig. SI-2). Results of the isotopic and hydrochemical investigations showed that the mainstem dilutes the nitrate contamination from wastewater inputs in tributaries, which is well defined by plotting isotopic and hydrochemical data of the Danube and its tributaries together (Figs. 6a, b, SI-7).

The PCA plots clearly show that persistent organic chemicals (PFOA and DNP) derived from urban and industrial wastewaters accumulate in the Danube mainstem. The negative correlation of persistent organic compound PFOA with the *δ*^18^O in river water (Fig. 5) is indicative of reduced biological processes in the Upper Danube River due to the lower water temperatures. Sulfamethoxazole is widely used as a veterinary drug and being often detected in the soil fertilized with the manure^40^. Therefore, the positive correlation of sulfamethoxazole with water *δ*^18^O (Fig. 5) is indicative of manure inputs with the lowland tributaries in the Lower Danube.

### Summary and recommendations

Our results revealed that tributary dilution and water origin are key considerations when interpreting pollutant data obtained from large river surveys. We observed that snowmelt-derived water fractions from the Inn River controlled not only Danube water chemistry but also dilution of pollutants for several hundred kilometers and influencing nitrate processes in the Danube. This study showed that although a large river system has complex mixtures of water and nitrate sources which are often localized in origin and hence cannot be directly source traced, the larger riverine systems have the advantage of slower integrated responses to e.g., changes in hydrological conditions, which allows the application of stable isotopes to evaluate ranges of sources and processes. To observe and better quantify these processes, like in-situ assimilation or nitrification a higher sampling frequency, monthly and seasonally targeted diurnal monitoring approaches would be required. Such intensive basin-wide monitoring stations could be combined with national monitoring stations and sampling for major ions and other chemical parameters. We found that tracers like CECs improved the interpretation of hydrological processes (e.g., water transit time) and supported the tracing of the nitrate sources, and the lack of biogeochemical processes. Our study represents a first-order synoptic approach that can be adapted to other large watersheds to better improve our understanding of dilution and mixing processes and to better focus remediation efforts, which are important for decision making regarding transboundary water management strategies.

## Method

### Study area

The Danube River has 27 large and ca. 300 small tributaries, but the main tributaries and their average discharges are the Sava River (1564 m^3^/s), Tisza River (794 m^3^/s), Inn River (738 m^3^/s), and Drava River (577 m^3^/s)^2^. Geographically and hydrologically, the DRB is divided into: Upper (from the river source to Devin Gate), Middle (from Devin Gate to Iron Gate II) and the Lower (downstream Iron Gate II) Danube. The mean annual discharge of the Danube River is relatively constant but peaks in March and early summer with a low-flow period in late summer^7^. Precipitation in the basin ranges from 1010 mm/yr in highlands to 600 mm/yr in lowlands^41^. Water residence time in the Upper Danube is 1–3 years^27,28^. The Danube is regulated by > 700 dams and weirs with the largest hydropower operations at Iron Gate Dam I and II which cause a pronounced shift from a flowing riverine to an impounded lake system reaching up to 200–300 km upstream^42^.

Predominant N pollution sources and pathways to the Danube River are groundwater inputs (44%) and wastewater discharges (23%). The N inputs into the DRB from the agricultural sector accounts for ca. 65% by overland runoff and groundwater, mainly due to application of mineral fertilizers (55%), followed by manure (37%) and ammonia (8%), whereas urban settlements contribute ca. 35%^34^.

### Data, sampling and analysis

Data on ion concentrations (Ca^2+^, Mg^2+^, Na^+^, NH_4_^+^, Cl^-^, SO_4_^2−^ and NO_3_^−^), isotopes of nitrate (*δ*^18^O-NO_3_ and *δ*^15^N-NO_3_), water (*δ*^18^O and *δ*^2^H) and CECs (carbamazepine, caffeine, sulfamethoxazole, perfluorooctanoic acid (PFOA) and 2,4-dinitrophenol (DNP)) were obtained during the 4th synoptic Joint Danube Survey (JDS4) conducted by the ICPDR from 29 June to 9 July 2019. No significant rain events occurred during this summer sampling campaign. CECs selection (Table SI-1) were based on their known origin, physicochemical properties, removal capacity in wastewater treatment plants (WWTPs) in the DRB^11^ and their widespread occurrence in river waters. CECs data were from two studies^43,44^ and are available at the NORMAN Occurrence Database (https://www.norman-network.com/nds/empodat/chemicalSearch.php). Details about chemical analysis and quality control method for CECs are described in previous publication^11^.

Dissolved oxygen data were obtained from the ICPDR national surveys, which were conducted between 01 and 23 July 2019 at the same sampling locations as JDS4. Long-term stable isotope data for watershed precipitation inputs for Vienna (198 m a.s.l, Austria) and Grimsel (2164 m a.s.l, Switzerland), and stable isotope data for the Danube River at Engelhartszel (Austria) were from the International Atomic Energy Agency (IAEA) Global Network of Isotopes in Precipitation (GNIP) and Rivers (GNIR) (https://nucleus.iaea.org/wiser). The isotopic composition in precipitation at Grimsel is considered to be representative for the high elevation European stations in the DRB^45^. Discharge data from the stations Neu-Ulm and Passau-Ingling were from the Bavarian State Office for the Environment and for Achleiten-Donau from the Federal Ministry of Agriculture, Regions and Tourism, Austria.

In total, 29 river water samples were taken along a 2581 km stretch of the Danube River and 14 water samples from the mouths of the most important tributaries (Fig. 1). Water samples for *δ*^18^O and *δ*^2^H analysis were filtered through 0.45 μm cellulose syringe filters and stored in 60 mL high density polyethylene (HDPE) bottles without headspace. Nitrate, ammonia, and nitrate isotope samples were collected in 110 mL HDPE bottles, filtered through 0.45 μm nylon syringe filters, and preserved by 1 mL of 2.5 mM sulfanilic acid in 10% HCl per 100 mL of sample. Water samples for ion analysis were filtered through 0.45 μm cellulose syringe filters and were acidified with nitric acid for cation analysis. Stable isotopes of water and ion compositions were analyzed in the IAEA/FAO Soil and Water Management & Crop Nutrition Laboratory, Seibersdorf, Austria. Ion compositions were analyzed using an Eco IC Metrohm with the detection limit is 0.1 mg/L. Water isotopes were analyzed using Picarro L2130*i* with the precision of ± 0.1 ‰ for *δ*^18^O and ± 0.5 ‰ for *δ*^2^H. Nitrate and ammonia were analyzed with an analytical error of 0.5 mg/L and 0.03 mg/L, respectively, using a discrete analyser (AQ1, Seal Analytical, Germany) in the IAEA Isotope Hydrology Laboratory, Vienna, Austria. Nitrate isotopes were analyzed using the Titanium (III) Chloride method^46^ on an Isoprime-100™ continuous-flow isotope-ratio mass spectrometer with the precision of ± 0.4 ‰ for *δ*^15^N-NO_3_ and *δ*^18^O-NO_3_ in the IAEA Isotope Hydrology Laboratory, Vienna, Austria.

### Calculations

Mixing of Danube and Inn River water was quantified using an isotope mass balance, where*:*

1 $$\delta^{18} O_{{\text{I}}} \cdot {\text{x}}_{{\text{I}}} + \delta^{18} {\text{O}}_{{\text{D}}} \cdot x_{D} = \delta^{18} {\text{O}}_{{{\text{DI}}}}$$

with *δ*^18^*O*_*I*_ = isotopic composition of the Inn River, x_I_ = mole fraction of Inn River water, *δ*^18^O_D_ = isotopic composition of Danube River water before mixing, x_D_ = mole fraction of Danube River water before mixing, *δ*^18^O_DI_ = isotopic composition of the mixed Inn and Danube River water (after confluent).

Considering that x_I_ + x_D_ = 1, the mole fraction of Inn River water after mixing is calculated with:

2 $$x_{{\text{I}}} = \left( {\delta^{18} O_{DI} {-}\delta^{18} O_{D} } \right){/}\left( {\delta^{18} O_{I} {-}\delta^{18} O_{D} } \right)$$

Statistical analysis was performed in R Core Team 2020 version 3.6.3. Principle component analysis (PCA) was done using the factoextra R package^47^ and was used to separate two principal components (PC1 and PC2) with hydrochemical parameters in relation to the Danube mainstem and its tributaries.

## Supplementary Information

Supplementary Information.
