# Supplementary material for: Nitrate sources and mixing in the Danube watershed: implications for transboundary river basin monitoring and management
Source: Sci Rep. 2022 Feb 9;12:2150. doi: 10.1038/s41598-022-06224-5 (PMC8828721; doi:10.1038/s41598-022-06224-5)
Supplement: Supplementary file 1 — Supplementary Information. [file 41598_2022_6224_MOESM1_ESM.docx]

**Supplementary Information**

**Nitrate sources and mixing in the Danube watershed: implications for transboundary river basin monitoring and management**

Halder J., Vystavna Y.*, Wassenaar L. I.

International Atomic Energy Agency, Department of Nuclear Sciences and Applications, Vienna International Centre, 1400 Vienna, Austria ([y.vystavna@iaea.org](mailto:y.vystavna@iaea.org) )

**Supplementary tables**

1. **Calculation of the isotope mass balance in the Danube River**

The Table SI-1 shows the calculated molar fraction of Inn River water (xI), calculated from values of Danube River water before mixing (D), the Inn River mouth (I), and Danube River water after mixing with Inn River water (DI). Daily average discharge data (Q) from the coherent sampling date were taken from the closest monitoring station (Passau-Ingling-Inn and Achleiten-Donau).

Table SI-1. Mass balance calculation of mixing Inn and Danube River water

|  | | | | **δ^18^O**  **(2019)** | **δ^2^H**  **(2019)** | **T**  **(2019)** | | **Q**  **(2019)** |
| --- | --- | --- | --- | --- | --- | --- | --- | --- |
| **Location** | **‰** | | | | **‰** | **°C** | **m^3^/s d** | |
| **JDS4–4 (D)** | | –10.8 | | | –76.1 | 27.6 |  | |
| **JDS4–5 (I)** | | –14.2 | | | –101.8 | 16.0 | 1140 | |
| **JDS4–6 (DI)** | | | –13.3 | | –95.1 | 18.5 | 1696 | |
| **xI (%)** | | | 74 | | 74 | 78 | 67 | |

The calculation method is presented in the main text of the manuscript in the Method section.

1. **Selected compounds of emerging concern**

Physicochemical properties of compounds of emerging concern (CECs) used in the study are presented in the Table SI-2.

Table SI-2. General physicochemical properties and application of selected CECs

| Compound | CAS | Formula | Water solubility (mg/L) | LogKow | Dissociation coefficient (pKa) | Application |
| --- | --- | --- | --- | --- | --- | --- |
| Sulfamethoxazole (SMX) | 723-46-6 | C_10_H_11_N_3_O_3_S | 610 | 0.89 | 1.6 (5.7) | Antibiotic |
| Gemfibrozil  (GEM) | 25812-30-0 | C_15_H_22_O_3_ | 11 | 4.77 | 4.5 | Antilipemic (lipid and blood regulation) |
| Valsartan  (VAL) | 137862-53-4 | C_24_H_29_N_5_O_3_ | 85 | 4 | 3.6 | Angiotensin II receptor blocker (blood pressure regulation) |
| Caffeine (CAF) | 1958-08-02 | C_8_H_10_N_4_O_2_ | 21600 | -0.07 | 14 | Stimulant |
| Carbamazepine (CBZ) | 298-46-4 | C_15_H_12_N_2_O | 18 | 2.45 | 13.9 | Anticonvulsant |
| Perfluorooctanoic acid (PFOA) | 335-67-1 | C_8_HF_15_O_2_ | 3300 | 4.81 | 1.3 (2.8) | Surfactant |
| 2-4 Dinitrophenol (DNP) | 51-28-5 | C_6_H_3_(OH)(NO_2_)_2_ | 2790 | 1.67 | 4.09 | Used in the manufacture of dyes, wood preservatives, and as a pesticide |

CECs concentrations in Danube mainstem and tributaries is in Table SI-3.

Table SI-3. Concentration of CECs in Danube mainstem and tributaries (ng/L). Data are taken from and are available at the NORMAN Occurrence Database (<https://www.norman-network.com/nds/empodat/chemicalSearch.php>).

| Site | R km | SMX | GEM | VAL | PFOA | 2-4-Dinitrophenol (DNP) | CAF | CBZ |
| --- | --- | --- | --- | --- | --- | --- | --- | --- |
| **Danube mainstem** | | | | | | | | |
| Böfinger Halde | 2581 | 3.68 | 6.24 | 11.46 | 0.80 | 1.71 | 12.27 | 9.15 |
| Bittenbrunn 700m below power station | 2479.5 | 0.48 | 22.14 | 0.00 | 1.20 | 3.41 | 9.89 | 12.27 |
| Kelheim | 2415 | 1.00 | 24.56 | 18.14 | 1.35 | 3.11 | 6.18 | 7.83 |
| Niederalteich - Mühlau | 2276 | 1.12 | 0.00 | 0.00 | 1.44 | 3.53 | 10.69 | 28.07 |
| Jochenstein | 2205 | 0.00 | 0.00 | 0.00 | 2.76 | 3.25 | 1.96 | 2.64 |
| Enghagen | 2121 | 0.43 | 5.25 | 0.00 | 3.06 | 3.41 | 7.97 | 16.43 |
| Oberloiben | 2007 | 0.00 | 5.74 | 0.00 | 2.91 | 3.50 | 12.10 | 10.85 |
| Klosterneuburg | 1942 | 0.00 | 0.00 | 0.00 | 2.85 | 3.57 | 3.44 | 22.74 |
| Hainburg, upstream Morava | 1882 | 0.43 | 10.15 | 0.00 | 2.17 | 3.90 | 11.62 | 16.62 |
| Bratislava | 1868 | 0.00 | 8.69 | 0.00 | 2.49 | 2.89 | 35.45 | 12.12 |
| Čunovo, Gabcikovo resevoir | 1855 | 0.00 | 0.00 | 0.00 | 2.85 | 3.48 | 1.34 | 2.04 |
| Medvedov / Medve | 1806 | 0.00 | 6.78 | 0.00 | 3.74 | 5.29 | 4.67 | 5.28 |
| Gönyű | 1790 | 0.53 | 0.00 | 0.00 | 3.01 | 4.99 | 7.98 | 8.17 |
| Szob | 1707 | 0.00 | 13.77 | 0.00 | 2.34 | 3.26 | 23.12 | 8.73 |
| Budapest upstream - Megyeri Bridge | 1660 | 0.00 | 8.32 | 0.00 | 3.37 | 5.30 | 16.52 | 5.82 |
| Budapest downstream - M0 bridge | 1630 | 0.34 | 0.00 | 0.00 | 3.49 | 7.69 | 18.17 | 5.58 |
| Dunafoldvar | 1560 | 1.16 | 17.01 | 4.20 | 4.28 | 4.57 | 14.30 | 24.29 |
| Paks | 1532 | 0.77 | 8.17 | 5.90 | 4.23 | 4.40 | 23.29 | 21.98 |
| Baja | 1481 | 0.32 | 9.22 | 3.43 | 3.08 | 4.06 | 7.05 | 16.69 |
| Hercegszanto / Batina / Bezdan | 1434 | 0.00 | 8.45 | 5.34 | 3.25 | 3.00 | 4.90 | 21.14 |
| Ilok / Backa Palanka | 1300 | 0.57 | 7.82 | 0.00 | 2.79 | 3.79 | 7.64 | 15.37 |
| Downstream Pancevo | 1151 | 1.20 | 0.00 | 0.00 | 1.37 | 3.61 | 70.42 | 20.74 |
| Banatska Palanka / Bazias | 1073 | 0.43 | 0.00 | 0.00 | 2.03 | 5.94 | 17.81 | 3.99 |
| Upstream Timok (Rudujevac / Gruia) | 847 | 0.00 | 0.00 | 0.00 | 1.94 | 3.44 | 7.95 | 3.45 |
| Pristol / Novo Selo Harbour | 837 | 0.00 | 0.00 | 0.00 | 1.50 | 3.78 | 6.61 | 3.60 |
| Downstream Ruse/Giurgiu (Marten) | 488 | 0.64 | 0.00 | 0.00 | 1.57 | 2.22 | 5.02 | 3.52 |
| Chiciu/Silistra | 375 | 0.49 | 0.00 | 0.00 | 1.83 | 3.52 | 9.65 | 3.01 |
| Reni | 132 | 0.00 | 0.00 | 0.00 | 1.88 | 2.57 | 2.60 | 7.52 |
| Vilkova - Chilia arm/Kilia arm | 18 | 7.71 | 7.97 | 0.00 | 1.80 | 2.57 | 48.34 | 101.15 |
| **Tributaries** | | | | | | | | |
| Inn | 4 | 0.00 | 0.00 | 0.00 | 1.60 | 1.78 | 19.87 | 5.08 |
| Morava/Dyje | 17 | 8.72 | 0.00 | 2.87 | 3.21 | 7.17 | 8.03 | 19.62 |
| Morava | 79 | 5.01 | 0.00 | 3.86 | 2.73 | 4.68 | 8.76 | 19.49 |
| Morava | 1 | 2.86 | 0.00 | 0.00 | 2.66 | 2.90 | 26.60 | 14.99 |
| Moson Danube Arm | 0.1 | 1.71 | 21.31 | 0.00 | 3.47 | 4.81 | 17.29 | 14.54 |
| Vah | 1.5 | 0.00 | 15.43 | 0.00 | 2.94 | 4.46 | 13.56 | 9.81 |
| Hron | 1.7 | 0.91 | 36.06 | 0.00 | 0.91 | 1.23 | 9.08 | 25.09 |
| Ipeľ | 12 | 0.00 | 11.14 | 7.71 | 0.92 | 3.27 | 10.65 | 33.69 |
| Ráckevei-Soroksári | 59 | 0.00 | 0.00 | 8.90 | 3.55 | 4.26 | 9.24 | 20.97 |
| Drava | 5 | 0.00 | 0.00 | 3.48 | 0.95 | 5.34 | 27.89 | 13.68 |
| Tisza | 163 | 0.00 | 0.00 | 0.00 | 0.57 | 3.19 | 9.26 | 9.71 |
| Tisza | 1 | 0.37 | 6.38 | 0.00 | 0.73 | 2.40 | 10.84 | 29.05 |
| Sava | 729 | 0.00 | 21.89 | 7.93 | 1.18 | 4.63 | 5.96 | 8.97 |
| Sava | 205 | 1.56 | 11.47 | 16.29 | 0.92 | 4.45 | 25.60 | 10.35 |
| Sava | 7 | 0.00 | 0.00 | 3.42 | 1.63 | 2.79 | 10.53 | 6.72 |
| Velika Morava | 154 | 1.12 | 0.00 | 0.00 | 0.65 | 1.60 | 16.62 | 8.88 |
| Velika Morava | 1 | 1.09 | 0.00 | 0.00 | 0.50 | 1.21 | 9.48 | 7.47 |
| Timok | 0.2 | 1.28 | 0.00 | 0.00 | 0.50 | 1.07 | 12.39 | 5.01 |
| Iskar | 0.3 | 2.32 | 15.23 | 3.01 | 1.45 | 1.73 | 2.64 | 11.15 |
| Jantra | 1 | 0.00 | 0.00 | 0.00 | 0.80 | 3.68 | 4.19 | 1.95 |
| Russenski Lom | 0 | 0.51 | 0.00 | 0.00 | 0.71 | 5.38 | 22.56 | 3.85 |
| Prut | 0.5 | 0.00 | 0.00 | 0.00 | 0.00 | 0.79 | 1.19 | 0.96 |

**Supplementary figures**

1. **Isotopic and temperature variation in the Danube mainstem and its tributaries**

Data on spatial variation of *δ*^2^H in Danube mainstem and tributaries are presented in Figure SI-1. Data on spatial variation of d-excess and water temperature are presented in Figure SI-2.

**Figure SI-1**. Longitudinal isotopic values (*δ*^2^H) in the Danube mainstem and tributaries.

1. **(b)**

**Figure SI-2.** Longitudinal *d*-excess value (a) and water temperature (b) in the Danube mainstem and tributaries.

1. **Principle component analysis**

Results of principal component analysis (PCA) show the relationships between chemical, isotopic and CECs variables in Danube mainstem and tributaries in Figure SI-3.

|  |
| --- |
| 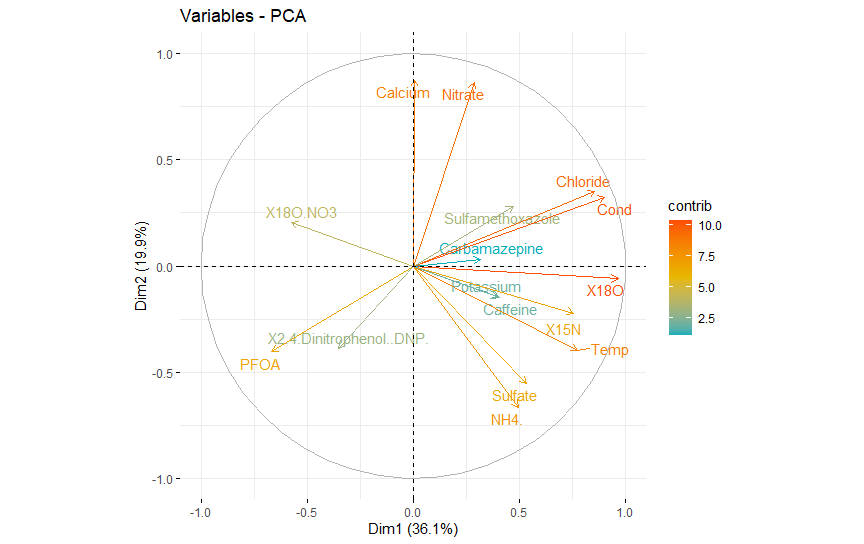  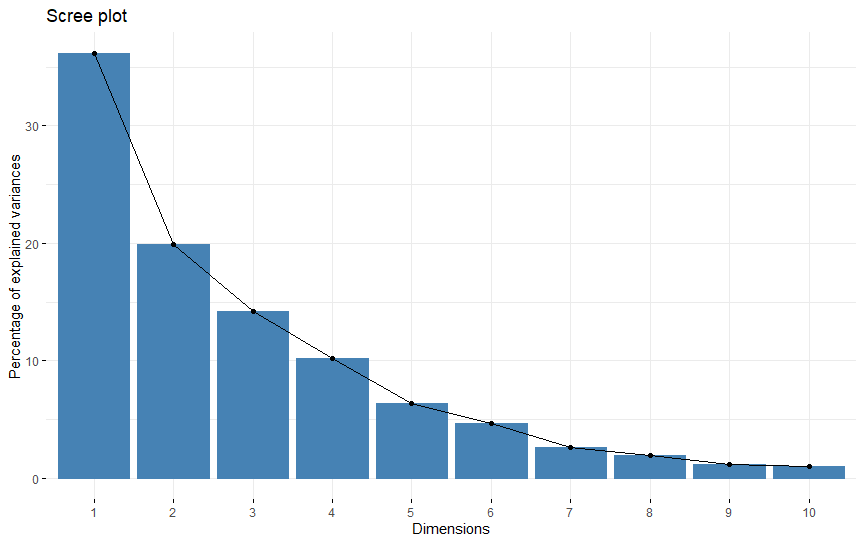 |
| 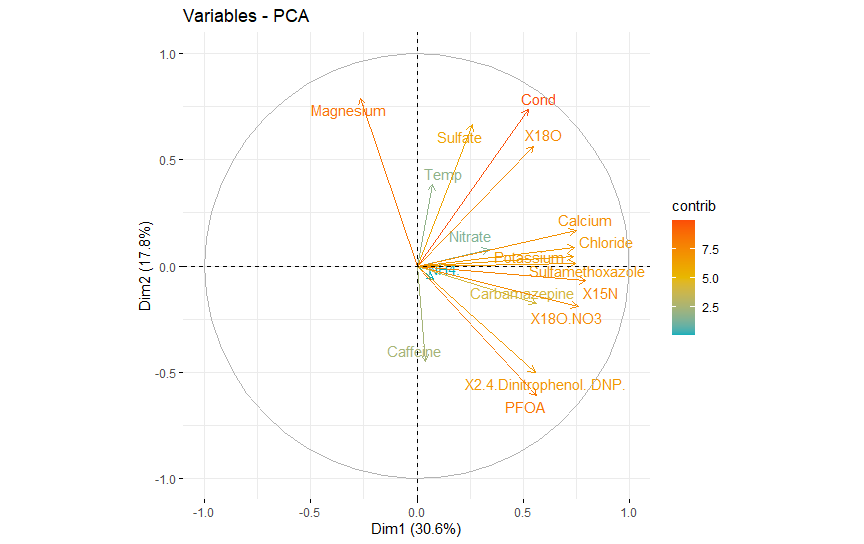  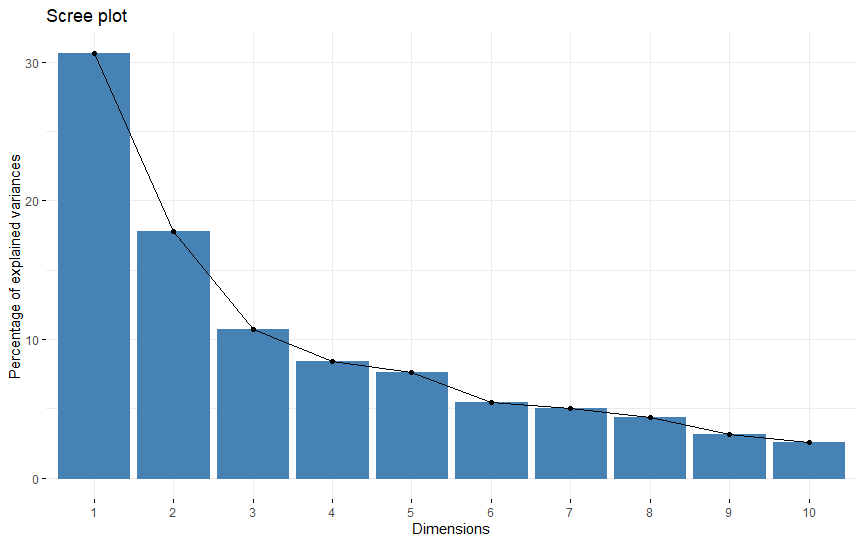 |

**Figure SI-3.** PCA and scree plots explain the relationships between chemical, isotopic and CEC variables in (a) Danube River and (b) its tributaries.

**3. Chemical parameters variations in the Danube mainstem and its tributaries**

The boxplots in Figure SI-4 explain the statistical difference between ions concentration in Danube mainstem and tributaries.

**
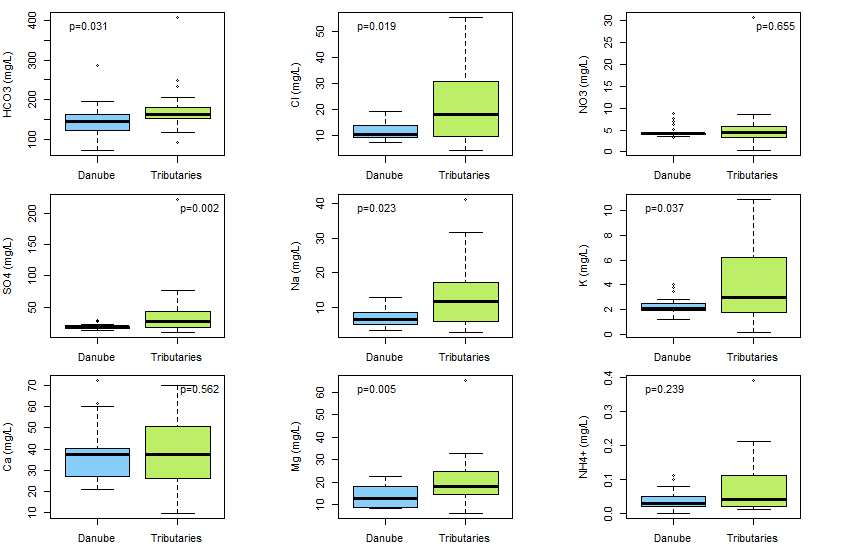
**

**Figure SI-4.** Boxplot explains the difference in ions concentration in Danube mainstem and tributaries.

Longitudinal values of ammonium concentration in the Danube mainstem and tributaries are presented in Figure SI-5 and these values for dissolved oxygen are presented in Figure SI-6.

**Figure SI-5.** Longitudinal values of ammonium concentration in the Danube mainstem and tributaries.

**Figure SI-6.** Longitudinal values of dissolved oxygen concentration in the Danube mainstem and tributaries.

Scree plot to support the PCA results (Figure 5a,b) is presented in the Figure SI-7.


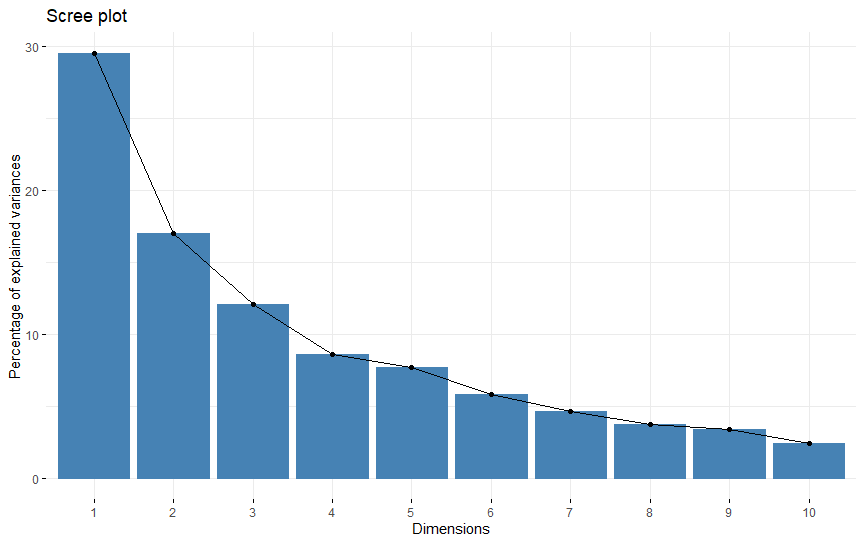


**Figure SI-7.** Scree plot showing the relationships between chemical, isotopic and CEC variables in Danube River and its tributaries presented as a PCA in Figure 5 (a,b).
